# Size and Structure of Empty and Filled Nanocontainer Based on Peptide Dendrimer with Histidine Spacers at Different pH

**DOI:** 10.3390/molecules26216552

**Published:** 2021-10-29

**Authors:** Valeriy V. Bezrodnyi, Sofia E. Mikhtaniuk, Oleg V. Shavykin, Igor M. Neelov, Nadezhda N. Sheveleva, Denis A. Markelov

**Affiliations:** 1 St. Petersburg State University, 7/9 Universitetskaya nab., 199034 St. Petersburg, Russia; v.v.bezrodniy@mail.ru (V.V.B.); shevelevann@gmail.com (N.N.S.); markeloved@gmail.com (D.A.M.); 2 St. Petersburg National Research University of Information Technologies, Mechanics and Optics (ITMO University), Kronverkskiy pr. 49, 197101 St. Petersburg, Russia; mikhtanyuk@mail.ru; 3 Tver State University, Zhelyabova St., 33, 170100 Tver, Russia

**Keywords:** peptide dendrimer, pH responsive spacers, molecular dynamics simulation, histidine–histidine pairing, nanocontainer capacity

## Abstract

Novel peptide dendrimer with Lys-2His repeating units was recently synthesized, studied by NMR (Molecules, 2019, 24, 2481) and tested as a nanocontainer for siRNA delivery (Int. J. Mol. Sci., 2020, 21, 3138). Histidine amino acid residues were inserted in the spacers of this dendrimer. Increase of their charge with a pH decrease turns a surface-charged dendrimer into a volume-charged one and should change all properties. In this paper, the molecular dynamics simulation method was applied to compare the properties of the dendrimer in water with explicit counterions at two different pHs (at normal pH with neutral histidines and at low pH with fully protonated histidines) in a wide interval of temperatures. We obtained that the dendrimer at low pH has essentially larger size and size fluctuations. The electrostatic properties of the dendrimers are different but they are in good agreement with the theoretical soft sphere model and practically do not depend on temperature. We have shown that the effect of pairing of side imidazole groups is much stronger in the dendrimer with neutral histidines than in the dendrimer with protonated histidines. We also demonstrated that the capacity of a nanocontainer based on this dendrimer with protonated histidines is significantly larger than that of a nanocontainer with neutral histidines.

## 1. Introduction

Dendrimers are regular hyperbranched molecules. Classical dendrimers have a single core, many branched repeating units of the same structure and a large number of terminal groups which can be functionalized. Dendrimers with different chemical structures, for example, polyethyleneimine (PEI), polyamidoamine (PAMAM) and carbosilane dendrimers have been synthesized during the last 40 years [1,2,3,4,5]. Due to their unique properties, they are used in many industrial and biomedical applications [6,7,8,9,10,11,12,13,14,15]. At the same time, synthetic dendrimers are rather toxic. Dendrimers based on branched amino acids units are more biocompatible.

Lysine dendrimers (consisting of only one type (lysine) of amino acid residue) were synthesized in late 1970s [16]. The properties of lysine dendrimers as well as their applications have been studied in many papers [17,18,19,20,21,22,23,24]. More complex peptide dendrimers consisting of various amino acids residues have been synthesized also [25]. They are used as multiple antigen peptides (MAPs) [26], and in many other applications including drug and gene delivery [27,28,29,30,31,32,33,34,35,36,37,38,39,40,41,42,43,44,45,46,47]. Lysine dendrimers and other dendrimers with terminal amino acid residues (for example, arginine or histidine) are also referred to as peptide dendrimers [48,49,50].

Recently, new lysine-based peptide dendrimers with dipeptide (2Gly, 2Lys, 2Arg and 2His) spacers between their branching points and lysine terminal groups have been synthesized and studied by NMR [35,36,37]. The possibilities of using these dendrimers for siRNA delivery have been studied as well [38,39]. Molecular dynamic simulation has also been applied to some of these dendrimers [40,41]. It has been shown that dendrimers with neutral and charged dipeptide insertion (2Gly and 2Lys, correspondingly, having Lys-2Gly and Lys-2Lys repeating units) have different size and other properties. Comparison of local mobility in the Lys-2Lys and Lys-2Arg dendrimers (with inserted 2Lys and 2Arg dipeptide spacers, correspondingly) showed that the mobility of the side groups in Lys-2Lys is close to the mobility of the terminal ones. However, for the Lys-2Arg dendrimer, it was found that the mobility of the side groups is close to the mobility of the inner main chain groups [36]. The authors suggested that the latter result was due to the effect of arginine–arginine pairing [51,52]. However, in our recent paper [41], it was shown that the pairing effect is small and this difference arises from the semi-flexibility effect associated with the different contour lengths from NMR active CH${}_{2}$-N groups to the end of lysine or arginine side chain in the spacers.

The peptide dendrimers synthesized by us differ from the traditional lysine dendrimers of Denkewalter [16] because they have a significantly lower branching asymmetry. Indeed, common lysine dendrimers have a branching asymmetry of 7:3 = 2.33. In some works, fears have been expressed that this asymmetry of branching can lead to an increase in the anisotropy of the lysine dendrimers themselves and a decrease in the free volume available for guest molecules [53]. The peptide dendrimers considered in our work have a branching asymmetry of 13:9 = 1.44 (in this case, the long spacers between branching points are approximately twice elongated, and the short ones are three times longer). In other words, in our dendrimers, the branching asymmetry is 1.6 times less than in traditional lysine dendrimers and it differs from the asymmetry of PAMAM dendrimers by less than 1.5 times.

As follows from our work on computer modeling of lysine and peptide dendrimers [44,45,47], such small branching asymmetry does not affect the structural characteristics of dendrimers and their properties are close to those of symmetric dendrimers. Therefore, our new peptide dendrimers can be considered as almost symmetric. The lengthening of the spacers between their branching points leads not only to a significant decrease in the asymmetry of branching, but also to the potential possibility of increasing their capacity for guest molecules due to the possibility of greater swelling of these dendrimers in comparison with conventional lysine dendrimers.

The goal of the present paper is to compare the size, structure and histidine–histidine pairing in a novel peptide dendrimer with Lys-2His repeating units in neutral and fully protonate states (i.e., at normal and low pH conditions, correspondingly) using molecular dynamics simulation. The size, internal structure and composition of the complex of the dendrimer with short bioactive peptides at different pH were also evaluated.

## 2. Materials and Methods

**Figure 1:**
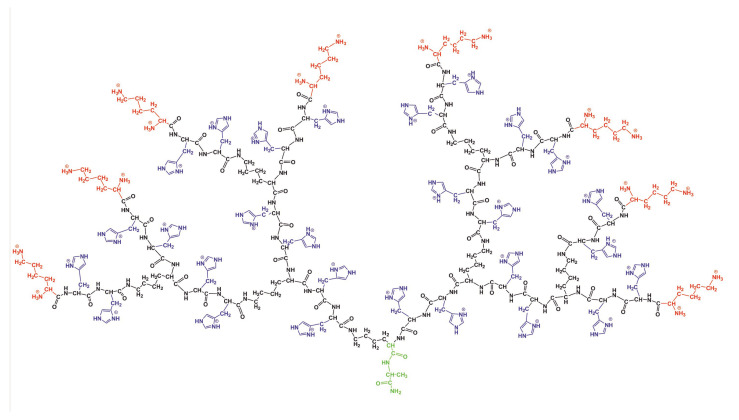
The chemical structure of the Lys-2Hisp dendrimer at low pH conditions. The dendrimer core is marked by green color, the backbone by black color, side segments by violet color and terminal lysines by red color.

In the paper, we used a the full atomic model of the lysine (Lys) dendrimer of the second generation with double histidine amino acid residues (2His) inserted as spacers between its neighboring branching points (see Figure 1). Thus, the repeating units of the studied dendrimer are Lys-2His. The two conditions were considered: (i) normal pH, when the histidine amino acid residue is neutral (His), and (ii) low pH, when histidine is fully protonated. In the latter case, we used the name Hisp for this. In the rest of the paper, we will call the dendrimer with neutral histidines as the Lys-2His dendrimer and the dendrimer with protonated histidines as the Lys-2Hisp dendrimers or simply Lys-2His and Lys-2Hisp. The characteristics of these dendrimers are provided in Table 1. The dendrimers in Figure 1 has an alanine-lysine core, a backbone formed by a branched main peptide chain with Lys-2His or Lys-2Hisp repeating units, and terminal lysine residues. The number of histidines $N_{ins}$ is 28 and the number of terminal NH${}_{3}^{+}$ groups N${}_{end}$ is 16. Both dendrimers were placed in a cubic box with periodical boundary conditions filled by water and Cl${}^{-}$ counterions. The bare charge of the dendrimer ($Q_{bare}=+16$ for Lys-2His and $Q_{bare}=+44$ for Lys-2Hisp) is the sum of the charge of terminal lysine groups ($Q_{end}=+16$ for both of them) and the charge of double histidine spacers ($Q_{ins}=0$ and $Q_{ins}=+28$ for Lys-2His and Lys-2Hisp, correspondingly). The parameters for the simulated systems are given in Table 1.

The molecular dynamics simulation was carried out by the Gromacs molecular simulation package [54]. The AMBER-99SB-ILDN force field [55] was chosen to describe all interactions in the system because it was tested by us earlier for similar highly branched systems (lysine dendrimers and dendrigrafts of different generations). More detailed description of the model and parameterization of branched lysine amino acid residues is given in [22]. The initial equilibration consisted of the same steps as in our previous simulations (energy minimizations in vacuum and in water and additional equilibration by the MD method with small integration steps). The long productive MD simulation (8 runs of 250 ns each) was carried out in the NPT ensemble. The constant temperature and the constant pressure were implemented via the Nose–Hoover thermostat [56] (with the time constant τ=0.4 ps) and the Parrinello–Rahman barostat [57] (with τ=0.5 ps and with the compressibility of water, weakly dependent on temperature [58]). We analyzed the simulation results using programs and procedures that were described in our previous papers on the simulation of linear polymers and polyelectrolytes [59,60,61,62,63,64], AFM of linear biopolymers [65], cyclization of linear peptides [65], polymers brushes [66], dendrimers and hyperbranched polymers in shear and elongational flows [67,68].

## 3. Results

### 3.1. The Global Characteristics

**Figure 2:**
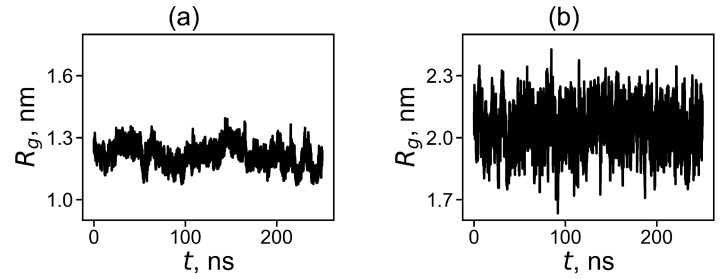
The time evolution of the instant radius of gyration $R_{g}$ at 310 K for (**a**) Lys-2His and (**b**) Lys-2Hisp.

The size and shape of the dendrimer are important for the practical applications of dendrimer in drug and gene delivery. There are various ways to estimate the size of a dendrimer. The calculation of the instant radius of gyration $R_{g}$ is one of them:

(1) $$R_{g}={\left(\frac{1}{M}\sum_{i}m_{i}r_{i}\right)}^{1/2},$$

where $M,\;m_{i}$ is the molecular masses of the dendrimer as a whole and of its *i*-th atom correspondingly and $r_{i}$ is the distance from the *i*-th atom to the center of mass of the dendrimer. The time evolution of $R_{g}$ for the dendrimer with neutral and protonated histidine amino acid residues are shown in Figure 2. It is easy to see that the instant size and fluctuations of the size are significantly higher for the protonated Lys-2Hisp dendrimer than for the neutral Lys-2His dendrimer.

Characteristics of the size of the Lys-2His and Lys-2Hisp dendrimers are collected in Table 2. As can be seen, for the Lys-2Hisp dendrimer the value of each characteristic is greater than for the Lys-2His dendrimer. We will refer to particular characteristics from this table in the rest of the paper.

**Figure 3:**
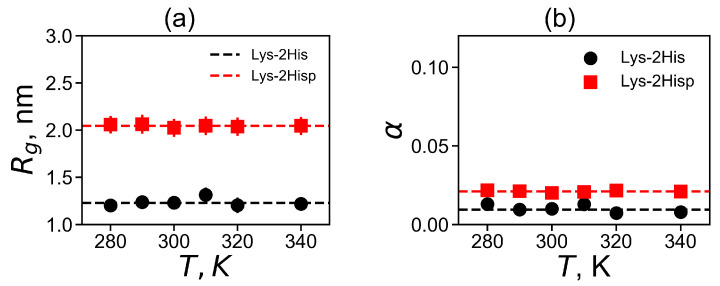
The temperature dependencies of (**a**) the mean-squared radius of gyration $R_{g}$ and (**b**) the shape anisotropy α. All data for Lys-2His and Lys-2Hisp.

In Figure 3a, we plot the average value of $R_{g}$ for each temperature. The average $R_{g}$ is significantly larger for the Lys-2Hisp dendrimer with protonated histidines, and it is close to $R_{g}$ or dendrimers with similarly charged 2Lys or 2Arg spacers in dendrimers with repeating units Lys-2Lys and Lys-2Arg correspondingly. The size of Lys-2His with neutral histidine spacers is close to $R_{g}$ for a dendrimer with neutral double glycine spacers (with Lys-2Gly repeating units).

Dendrimer shape is another important characteristic for applications. We used the asphericity parameter α for the evaluation of the dendrimer shape [69,70,71]:

(2) $$\alpha =1-3\frac{I_{x}I_{y}+I_{x}I_{z}+I_{y}I_{z}}{{\left(I_{x}+I_{y}+I_{z}\right)}^{2}}$$

where $I_{x}$, $I_{y}$ and $I_{z}$ are the principal (characteristic) axes of the inertia tensor. The two limits α=1 and α=0 correspond to an infinitely elongated rod and an ideal sphere, respectively. Dendrimers of small generations usually have ellipsoidal shape, however, an increase of the generation number leads to the more and more spherical shapes of dendrimers. The asphericity parameter α at the temperature of 310 K is presented in Table 2). For both dendrimers, α is very small, therefore their shape is close to spherical. At the same time, Lys-2His has a more spherical shape than Lys-2Hisp.

**Figure 4:**
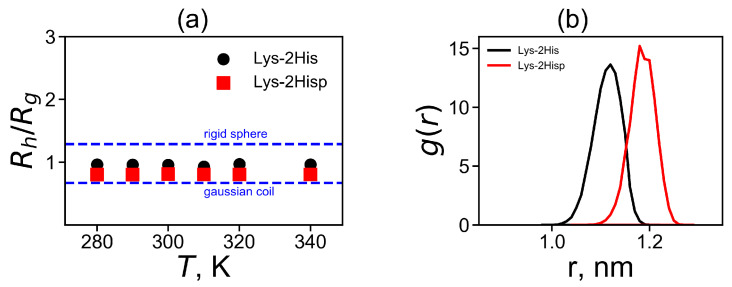
(**a**) The temperature dependences of the characteristic ratio $R_{h}$ (obtained by the Kirkwood approximation) to the radius of gyration $R_{g}$ and (**b**) the distribution g(d) of a spacer length *d* between the neighboring branching points of the dendrimers.

As the asphericity of our dendrimers at both pHs is small the diffusion of the Lys-2His and Lys2Hisp dendrimers in the solution can be described as the diffusion of a spherical particle with the Stokes radius. The coefficient of translational diffusion of the center of mass of dendrimer can be used for the calculation of the Stokes radius (hydrodynamic radius $R_{h}$), which is another characteristic of the dendrimer size. Another method for calculating this characteristic is to use the Kirkwood approximation [72,73]:

(3) $$R_{h}^{-1}={\langle r_{ij}^{-1}\rangle }_{i\neq j},$$

where $r_{ij}$ is a distance between two heavy atoms *i* and *j*. The additional characteristic is the characteristic ratio of $R_{h}$ and $R_{g}$ shown in Figure 4a. It is easy to see that for both Lys-2His and Lys-2Hisp dendrimers the characteristic ratio $R_{h}/R_{g}$ does not depend on temperature. Two theoretical limits [74] of ratio $R_{h}/R_{g}$ are a Gaussian coil (lower limit) and an impenetrable rigid sphere (upper limit). They are depicted in Figure 4a. The ratio $R_{h}/R_{g}$ is smaller for Lys-2Hisp and in this case it is closer to the Gaussian coil.

There are other characteristics of size as well. For example, sometimes it is important to know the position of the dendrimer–water interface. This interface could be evaluated from the outer boundary position of the dendrimer, i.e., as the radial distance from the center of the dendrimer to its spherical surface in solvent. In many simulations of dendrimers, it was shown that the behavior of dendrimers is similar to that of a dense sphere. For this model the outer boundary can be estimated as $\sqrt{5/3}R_{g}$ (see Table 2). Furthermore, the outer boundary of the dendrimer can be calculated from the position of the slip plane (the effective radius $R_{max}$), i.e., the boundary between the charged dendrimer surface and the diffusion layer of the salt solution. Later, we will more accurately determine the effective radius $R_{max}$ from the electrostatic properties of the dendrimer. Table 2 demonstrates that $\sqrt{5/3}R_{g}$ and $R_{max}$ are larger for Lys-2Hisp, than for Lys-2His.

The average position of the terminal groups $R_{e}$ of the dendrimer is another way to evaluate this boundary. $R_{e}$ can be calculated as the mean square radial distance from the center of the dendrimer to the nitrogen atoms in the terminal NH${}_{3}^{+}$ groups:

(4) $$R_{e}={\left(\frac{1}{N_{t}}\sum_{i=1}^{N_{t}}r_{i}^{2}\right)}^{1/2}$$

Here, the summation is performed over all nitrogen atoms in the terminal NH${}_{3}^{+}$ groups. The values of $R_{e}$ are provided in Table 2. The average position of the terminal groups $R_{e}$ is again larger for Lys-2Hisp, than for Lys-2His. This is due to the different spacer length in both dendrimers, because the contour path from the dendrimer center to the ends consists of the spacers. In order to illustrate the effect, we calculated and plotted in Figure 4b the distributions of the spacer length. It is easy to see that the distribution for the Lys-2Hisp dendrimer is shifted towards longer length of the spacer and is narrower due to electrostatic repulsion of protonated histidines in its spacers.

### 3.2. The Local Structure

#### 3.2.1. The Spatial Symmetry and Atomic Distributions

**Figure 5:**
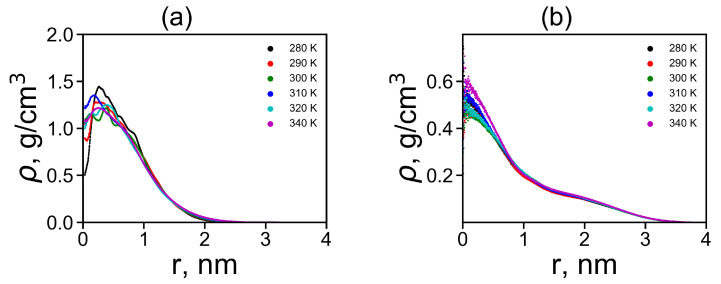
The radial distribution function of density for dendrimers at different temperatures: (**a**) Lys-2His and (**b**) Lys-2Hisp.

Using the spherical symmetry of the system, we can consider the radial density distribution function of the atomic density ρ(r) relative to the center of mass of the dendrimer:

(5) $$\rho \left(r\right)=\frac{1}{4\pi r^{2}}\sum_{i=1}^{N}m_{i}\delta \left(r-r_{i}\right)$$

where δ is the Dirac delta function, *r* is the radial distance from the center of mass of the molecule and $r_{i}$ is the radial distance from *i*-th atom to the center of mass of the molecule. We plotted the radial distributions ρ(r) in Figure 5a,b at different temperatures. Lys-2His has ~2 times denser inner region of the dendrimer, and a shorter tail of this distribution at large distances *r* from the dendrimer center, which is consistent with the smaller size of this dendrimer with neutral 2His spacers. The functions of ρ(r) for Lys-2His dendrimer is close to that of the Lys-2Gly dendrimer with similar neutral 2Gly spacers. For the Lys-2Hisp dendrimer with protonated 2Hisp spacers this function is close to that of the Lys-2Lys [40] and Lys-2Arg [41] dendrimers with similarly charged 2Lys and 2Arg spacers.

#### 3.2.2. Electrostatic Interactions

**Figure 6:**
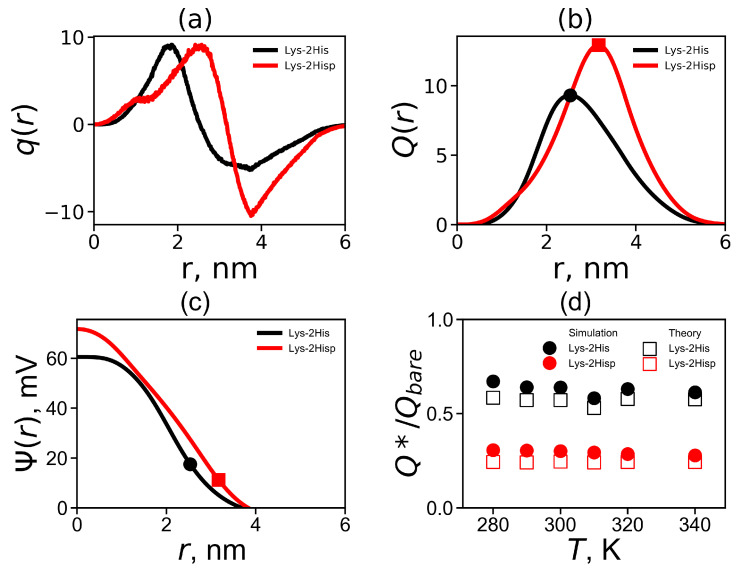
(**a**) The total charge distribution q(r), (**b**) the cumulative charge distribution Q(r), (**c**) the electrostatic potential Ψ(r) for Lys-2His and Lys-2Hisp at T=310 K and (**d**) the temperature dependencies of the relative effective charge $Q^{*}/Q_{bare}$.

The radial distribution of the total charge q(r) of the system calculated by summation over all charged atoms of the dendrimer and counterions is an important characteristic of a local structure of the dendrimer. Figure 6a shows the charge distribution q(r) at temperature 310 K. The curves q(r) for both Lys-2His and Lys-2Hisp dendrimers demonstrates classical double layer picture: containing a region q(r)>0 with a maximum at *r* close to the position of the charged ends of the dendrimer and area region with a negative value of q(r)<0 corresponding to a counterion cloud around the dendrimer. The value of the maximum is the same for both dendrimers but the minimum is less pronounced for the Lys-2His dendrimer. For the more compact Lys-2His dendrimer with neutral spacers, the positions of the maximum and minimum are shifted to shorter distances *r* from the center of the dendrimer.

The cumulative charge distribution Q(r) is the integral characteristic of charge distribution

(6) $$Q\left(r\right)=\int_{0}^{r}q\left(x\right)dx$$

Figure 6b compares the cumulative charge distribution Q(r) for Lys-2His and Lys-2Hisp at 310 K. The dots indicate the maxima on these curves. The position of these maxima is the effective radius $R_{max}$ (which was introduced above). The value of the cumulative charge distribution at these maxima corresponds to the effective dendrimer charge $Q^{*}$. The effective dendrimer charge $Q^{*}$ and the surface charge density $\sigma =Q^{*}/4\pi R_{max}^{2}$ [75] are represented in Table 3.

The electrostatic potential Ψ(r) can be found from the Poisson equation:

(7) $$\frac{d^{2}\psi \left(r\right)}{dr^{2}}+\frac{2}{r}\frac{d\psi (r)}{dr}=-kq\left(r\right)$$

where $\psi \left(r\right)=\left[e/k_{B}T\right]\Psi \left(r\right)$ is the dimensionless electrostatic potential, and $k=4\pi \lambda_{B}/dr$ is dimensionless factor (dr [nm]) is *r* with the Bjerrum length $\lambda_{B}$:

(8) $$\lambda_{B}=\frac{e^{2}}{4\pi \epsilon \epsilon_{0}k_{B}T}$$

where *e* is the elementary charge, ϵ is the relative dielectric permittivity of water (ϵ≈80), $\epsilon_{0}$ is the dielectric permittivity of vacuum, $k_{B}$ is the Boltzmann constant and *T* is the actual temperature. The electrostatic potential Ψ(r) is plotted in Figure 6c. Both curves have a different shapes: Ψ(r) for Lys-2His has a parabolic profile while for Lys-2Hisp the profile is closer to linear one. The values of the electrostatic potential at the effective radius corresponds to the ζ potential, i.e., $\zeta =\Psi (R_{max})$ (see Table 3). It is a good approximation for the ζ potential for a salt-free solution [76].

The theoretical model of soft sphere [77] was applied to the lysine dendrimer earlier. It was shown that this model describes the lysine dendrimer very well [23]. The theoretical effective charge $Q^{*}$ can be found from the following relation [77]:

(9) $$Q^{*}=\frac{R}{\lambda_{B}}\frac{1}{2\nu }\ln\left[\left(\frac{Q}{Q^{*}}-1\right)\left(\frac{D^{3}}{R^{3}}-1\right)\right]$$

where ν=3/5, $R=R_{max}$ is radius of the soft sphere (i.e., dendrimer), $Q=Q_{bare}$ is the bare charge of the dendrimer and $D=3^{1/2}a_{cell}/2$ is the cell radius, where $a_{cell}$ is the average size of a simulation cell (see Table 1). We compare the simulation and theoretical ratios $Q^{*}/Q_{bare}$ in Figure 6d. Good agreement with theory and simulation was obtained at all temperatures *T*. Note that the Lys-2His dendrimer has a smaller bare charge $Q_{bare}$ and a larger $Q^{*}/Q_{bare}$, than the Lys-2Hisp dendrimer.

**Figure 7:**
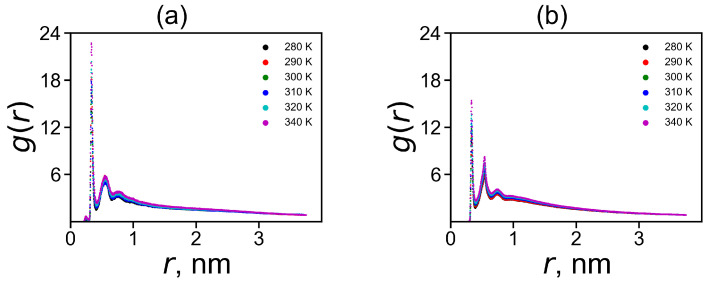
The radial distribution function of ion pairs between ions and charged terminal groups in (**a**) Lys-2His and (**b**) Lys-2Hisp at different temperatures.

A decrease in the charge $Q_{bare}$ to $Q^{*}$ is associated with the penetration of counterions into the dendrimer. The ion-pairs make one possible contribution to it. The ion pairs radial distribution function is shown in Figure 7. The characteristic peak in the region of 0.3 nm corresponds to the formation of ion pairs. The integration of the area under this peak gives a number of ion-pairs $\langle n_{ion\;pairs}\rangle$ in Table 3. However, not all ions that have penetrated into the dendrimer form ion pairs. Ions inside the dendrimer, that have not formed ion pairs, create osmotic pressure. Therefore, we can call such ions «osmotic», and their number can be easily calculated by the equation $\langle n_{ion\;osmotic}\rangle =Q-Q^{*}-\langle n_{ion\;pairs}\rangle$. The values $\langle n_{ion\;pairs}\rangle$ and $\langle n_{ion\;osmotic}\rangle$ for both dendrimers at 310 K are given in Table 3.

#### 3.2.3. The Hydrogen Bonds

Hydrogen bonds play important role in the stabilization of the structure of peptides and other biomolecules [78,79]. In this paper, we checked a number of intra-dendrimer hydrogen bonds in the Lys-2His and Lys-2Hisp dendrimers, a number of dendrimer–water hydrogen bonds for them and calculated their life-times. Here, we used here common geometrical criteria for hydrogen bond formation in MD simulations: the distance between the donor (D) and acceptor (A) atoms is less than 0.35 nm and the D-H-A angle is less than 30${}^{\circ }$[80]. The number of intra-dendrimer and dendrimer–water hydrogen bonds and their lifetimes at temperature T=310 K are shown for the Lys-2His and Lys-2Hisp dendrimer in Table 4. It is easy to see that there are about 13 intra-hydrogen bonds for the more compact dendrimer with neutral histidine and only one such hydrogen bond in the dendrimer with protonated histidines, while there are about 150 hydrogen bonds between each dendrimer and water.

**Figure 8:**
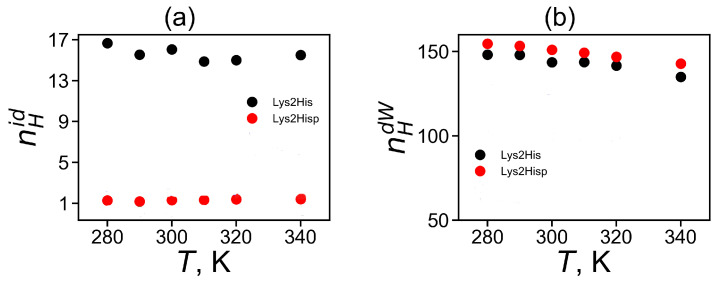
The average number of of (**a**) for intra-dendrimer and (**b**) for dendrimer-water hydrogen bonds as a function of temperature for Lys-2His and Lys-2Hisp.

The temperature dependence of the average number of intra-dendrimer $\langle n_{H}^{id}\rangle$ and dendrimer–water $\langle n_{H}^{dw}\rangle$ hydrogen bonds (HB) is represented in Figure 8a,b, correspondingly. It is clear that the number of intra-dendrimer hydrogen bonds for the dendrimer with neutral and protonated histidines practically does not change with temperature. At the same time, the number of intermolecular hydrogen bonds of the dendrimer with water molecules decreases with temperature similarly to that for other peptide dendrimers studied by us earlier [40,41]. The stability of hydrogen bonds could be estimated from the lifetime of these hydrogen bonds [81,82,83]. Among the various ways [82,83] to estimate the HB lifetime, we use the continuous hydrogen bond lifetimes [83]. Table 4 presents the intra-dendrimer HB lifetime $\tau_{BF}^{id}$ and the dendrimer-water HB lifetime $\tau_{BF}^{dw}$. Our results demonstrates that the lifetime $\tau_{BF}^{id}$ of intramolecular hydrogen bonds in the Lys-2His dendrimer with neutral (2His) spacers is near two times longer than the lifetime $\tau_{BF}^{dw}$ of intramolecular hydrogen bonds in the Lys-2Hisp dendrimer with protonated (2Hisp) histidines and about four times larger than lifetime of intermolecular hydrogen bonds of both dendrimers with water molecules.

### 3.3. The Imidazole and Guanidine Pairing

An unusual slowdown of the orientational mobility in 2Arg arginine spacers of the peptide dendrimer with Lys-2Arg repeating units has been found recently in the NMR experiment [36]. It was suggested that this is due to the arginine–arginine paring effect [51,52,84] previously studied for dimers and short arginine peptides. However, we have shown in our last paper that the pairing effect in the Lys-2Arg dendrimer is too small and another explanation of the slowdown was found [41]. In this paper, we focus on the possibility of pairing between imidazole groups [84] in the Lys-2His and Lys-2Hisp dendrimers and on comparing this with the guanidine pairing in the Lys-2Arg dendrimer obtained in our recent work. All possible pairs of plane groups in each system can be divided into pairs: (i) between neighboring His (from the same 2His spacer) and (ii) between non-neighboring His (belonging to different spacers). The matrix element $N_{pairs}\left(\theta ,r\right)$ is the number of imidazole–imidazole (or guanidine–guanidine) pairs at a distance *r* between their centers, and with the angle θ between the planes of these plane groups.

**Figure 9:**
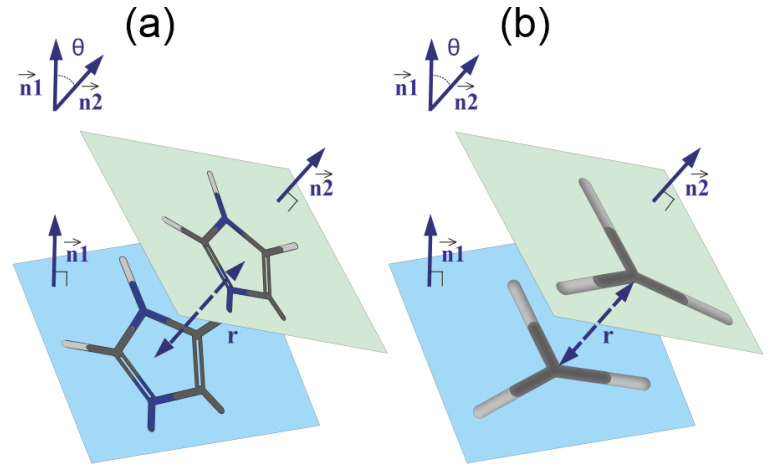
The schematic model of pairing of (**a**) imidazole–imidazole groups and (**b**) guanidine–guanidine groups. The geometrical parameters of the pairs: the distance *r* between the centers of the groups, the normal vectors ${\vec{n}}_{1}$ and ${\vec{n}}_{2}$ to the planes of the groups and the angle θ between these vectors are shown.

We studied pairing using the simple geometric model shown in Figure 9. Each plane group (imidazole or guanidine) lies completely in one plane. Therefore, the angle between these groups is the angle between the normals ${\vec{n}}_{1}$ and ${\vec{n}}_{2}$ to the planes of corresponding groups (see Figure 9). The coordinates (A,B,C) of the normal vector are the coefficients of the equation Ax+By+Cz+D=0 for the plane and, as is known, can be found by solving the equation

(10) $$Ax+By+Cz+D=\left|\begin{array}{ccc} x-x_{1} & x_{2}-x_{1} & x_{3}-x_{1}\\ y-y_{1} & y_{2}-y_{1} & y_{3}-y_{1}\\ z-z_{1} & z_{2}-z_{1} & z_{3}-z_{1} \end{array}\right|=0$$

where ${\left(x_{i},y_{i},z_{i}\right)}_{i=1,2,3}$ are the coordinates for three atoms forming the plane.

Let $\left(A_{1},B_{1},C_{1}\right)$ be the coordinates of the normal vector ${\vec{n}}_{1}$ to the plane of the first group (imidazole or guanidine). The coefficients $A_{1},B_{1},C_{1},D_{1}$ are obtained by solving the Equation (10) for the first group. Using the same scheme, we obtained the coordinates $\left(A_{2},B_{2},C_{2}\right)$ of the normal vector ${\vec{n}}_{2}$ to the plane of the second group (imidazole or guanidine). Then, the angle θ between the two planes can be found as the angle between the vectors of the normal to these planes

(11) $$\theta =\arccos\left(\frac{\left({\vec{n}}_{1},{\vec{n}}_{2}\right)}{|{\vec{n}}_{1}\left|\cdot \right|{\vec{n}}_{2}|}\right)$$

The distance *r* between two groups can be found as the distance between the centers of these groups. Thus, for each plane group, the distribution matrix $N_{pairs}$ can be calculated.

**Figure 10:**
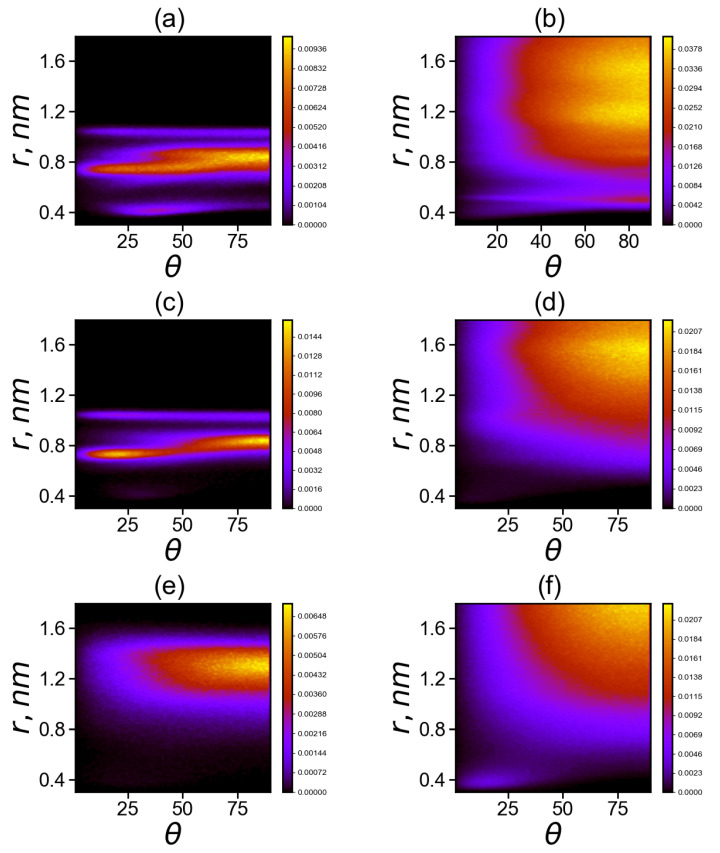
Two-dimensional maps of the distribution matrix $N_{pairs}$ (with matrix elements $N_{pairs}\left(\theta ,r\right)$) of imidazole–imidazole (or guanidine–guanidine) groups at T=310 K (**a**,**c**,**d**) for pairs between neighboring groups and (**b**,**d**,**e**) for pairs between non-neighboring groups in Lys–2His (**a**,**b**), Lys–2Hisp (**c**,**d**) and Lys–2Arg (**e**,**f**).

The matrices $N_{pairs}$ for neighboring and non-neighboring imidazole–imidazole pairs in Lys-2His and Lys-2Hisp are shown in the Figure 10a–d. The angle between the planes can range from 0 to 90 degrees. In both cases, a cut-off at a distance of 1.8 nm was chosen. Pairs located at larger distance are of no interest for analysis. The neighboring imidazole groups (from the same spacer) cannot go beyond this limit due to topological constraints. For comparison, we have added similar maps (see Figure 10e,f) for Lis-2Arg.

Comparison of two dimensional maps in Figure 10a,c,e for the neighboring His–His (a), Hisp–Hisp (c) or Arg–Arg (e) belonging to the same spacer (2His, 2Hisp or 2Arg, respectively) side groups shows that

(a) neighboring uncharged imidazole groups of histidines (His) form stable His–His pairs at three possible distances *r* between the centers of the imidazole groups: the first narrow region is located at distances *r* of about 0.4 nm (at angles between their planes from 15 to 80 degrees), the second is the wide range of distances *r* from 0.7 to 0.9 nm (with possible angles from 0 to 90 degrees) and the third narrow region is at a distance r=1.1 nm (with angles from 5 to 90 degrees) with a small number of pairs;

(c) neighboring protonated imidazole groups of histidines (Hisp) do not form pairs at distances (*r* about 0.4 nm); the region of Hisp–Hisp pairs from 0.7 to 0.9 nm become smaller than that for His–His pairs and the pairs in this region are more structured (at r=0.7 the angles are close to 20 degrees, and at r=0.9 nm the angles are close to 80 degrees) and the third narrow region with a small number of pairs at r=1.1 nm is similar to that for uncharged His;

(e) for neighboring charged guanidine groups of arginine pairs (Arg-Arg), there is only the region of existence of Arg–Arg pairs at large distances *r* from 1.1 to 1.5 nm with a wide range of possible angles from 30 to 90 degrees with the most probable distance *r* near 1.3 and angles from 70 to 90 degrees.

Comparison of two dimensional maps in Figure 10b,d,f for non-neighboring His–His (b), Hisp–Hisp (d), Arg–Arg pairs and (f) pairs belonging to different spacers (2His, 2Hisp or 2Arg, respectively) shows that

(b) non-neighboring uncharged imidazole groups of histidines (His) form stable His–His pairs at two possible distances *r* between their centers: the first narrow region is at distance *r* close to 0.4 nm (at angles between their planes from 10 to 90 degrees, mainly from 70 to 90) with a small number of pairs, the second one is a very wide unstructured region at distances from 0.6 to 1.6 nm;

(d) for non-neighboring protonated imidazole pairs (Hisp–Hisp), there is no the narrow region at *r* about 0.4 nm, but there is a wide region of existence of Hisp–Hisp pairs at distances *r* from 0.7 to 1.75 nm with a wide interval of possible angles from 25 to 90 degrees (with the most probable range of distances from 1.3 to 1.6 nm and angles from 65 to 90 degrees).

(f) for non-neighboring charged guanidine groups of arginine pairs (Arg–Arg), there are two regions: the first region is very narrow and compact (around *r* = 0.4 nm, with angles from 0 to 30 degrees; the second one is a wide region of existence of Arg–Arg pairs at distances *r* from 0.8 to 1.8 nm, and with a wide range of possible angles from 30 to 90 degrees (with the most probable distances *r* from 1.6 to 1.8 nm and angles from 70 to 90 degrees). It is interesting to note that this region does not exist for neighboring guanidine groups (belonging to the same spacer) in the Lys–2Arg dendrimer, and it appears in these dendrimers only for non-neighboring pairs.

Another important characteristic is the radial distribution obtained by averaging the $N_{pairs}$ over all possible orientational angles between two plane groups:

(12) $$n_{pairs}\left(r\right)=\int_{0}^{90^{\circ }}N_{pairs}\left(\theta ,r\right)d\theta$$

**Figure 11:**
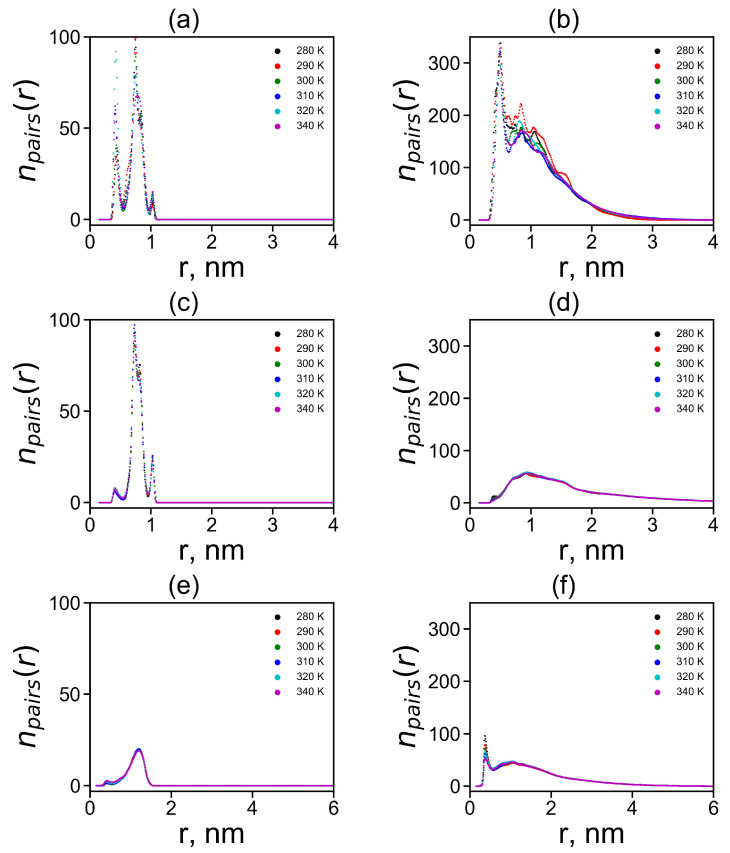
The radial distribution functions $n_{pairs}\left(r\right)$ of the centers of imidazole–imidazole (or guanidine–guanidine) groups at different temperatures (**a**,**c**,**d**) for pairs between neighboring groups and (**b**,**d**,**e**) for pairs between non-neighboring groups in Lys–2His (**a**,**b**), Lys–2Hisp (**c**,**d**) and Lys–2Arg (**e**,**f**).

The radial distributions for neighboring and non-neighboring pairs are shown in Figure 11 at different temperatures. It is easy to see that the results practically independ of temperature, with the exception of some relatively small irregular fluctuations in Figure 11b.

In the case of neighboring pairs of the studied plane groups, a sharp peak of $n_{pairs}$ exists in the region of the smallest possible distances r=0.4 nm between plane groups only for neutral His–His pairs. For charged Hisp–Hisp and Arg–Arg pairs, a peak at this distance also exists but its height is negligible. There is a more intense and broader second peaks for His–His and Hisp–Hisp pairs at distances *r* from 0.6 to 0.9 nm and a smaller third peak at distances close to r=1.1 nm. For Arg–Arg pairs, there is only a peak trace at r=0.4 and a rather wide and flat elevated area of the number of Arg–Arg pairs between r=0.4 and 1.5 nm.

In the case of non-neighboring pairs, a strong peak of $n_{pairs}$ in the region of the minimum possible distances r=0.4 nm is observed for neutral His–His pairs. For Hisp–Hisp pairs, the peak at this distance (r=0.4 nm) is negligible while for Arg–Arg non-neighboring pairs it exists but it is three times smaller than for His-His pairs. Thus, the largest first peak at r=0.4 nm for His–His pairs between non-neighboring groups is in Lys-2His (see Figure 11b). This means that in this case there is the largest number of non-neighboring pairs of plane groups.

We integrated the area under the first peak in all systems to estimate the average number of these closest pairs

(13) $$n_{p}=\int_{0}^{r_{cut}}\int_{0}^{90^{\circ }}N_{pairs}\left(\theta ,r\right)d\theta dr=\int_{0}^{r_{cut}}n_{pairs}\left(r\right)dr$$

where the upper limits were chosen to take into account the number of pairs under peak at r=0.4 nm. The choice of the integration limits corresponds to the restrictions chosen by the authors of other works [51,52,84].

**Figure 12:**
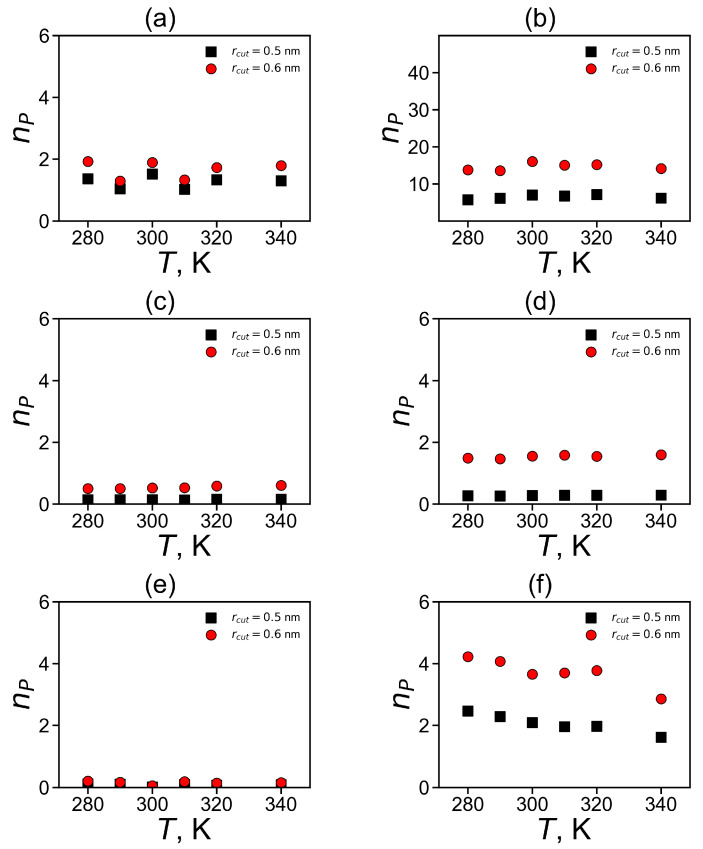
The average number of pairs for imidazole–imidazole (or guanidine–guanidine) groups at different temperatures (**a**,**c**,**d**) for pairs between neighboring groups and (**b**,**d**,**e**) for pairs between non-neighboring groups in Lys–2His (**a**,**b**), Lys–2Hisp (**c**,**d**) and Lys–2Arg (**e**,**f**).

The dependences of the number of pairs $n_{p}$ on temperature are shown in Figure 12. It is easy to see that for almost all types of the studied plain groups (His–His, Hisp–Hisp) there are practically no temperature dependences, with the exception of fluctuation due to the finite time of the simulation. There is a slight systematic decrease in $n_{p}$ for Arg–Arg pairs (see Figure 12f). Due to this reason, we compared the values of $n_{p}$ for different dendrimers only for T = 310 K.

It can be summarized that the total number of pairs $n_{p}$ of plane groups is the highest for non-neighboring pairs of non-charged (His) imidazole groups in Lys-2His dendrimer (see Table 5). This is due to the fact that the His groups in this dendrimer are uncharged and there is no need to overcome electrostatic interactions for pairing. It is interesting that the next highest number is for non-neighboring Arg–Arg pairs and then for non-neighboring Hisp–Hisp pairs. For neighboring pairs $n_{p}$ is the highest for His–His, and then for Hisp–Hisp pairs, and the smallest $n_{p}$ is for neighboring Arg–Arg pairs.

The pairing between neighboring plane groups belonging to the same spacer could influence only the local structure and mobility in the dendrimer. Thus, the most important results obtained in this work are the existence of the largest number of non-neighboring pairs in the Lys–2His dendrimer with neutral histidines (see Figure 10b, Figure 11b and Table 5), and a significant lower number of pairs in the Lys–2Hisp dendrimer with protonated histidines and in the Lys-2Arg dendrimer. Furthermore, only in the Lys–2His dendrimer pairing could affect both the local and global structure and mobility of the dendrimer.

At the same time, we found that all plane groups do not form permanent pairs that exist during the entire simulation time and pairs can be form and break up during the calculation. Therefore, the important characteristic of pairing in the dendrimers with different plane groups is also their lifetimes.

**Figure 13:**
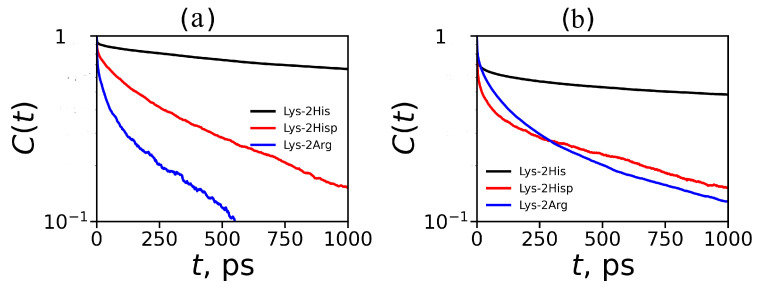
Autocorrelation functions $C_{pairs}\left(t\right)$ for imidazole–imidazole groups for Lys–2His and Lys–2Hisp (or guanidine–guanidine for Lys–2Arg) at T=310 K (**a**) for pairs between neighboring groups and (**b**) for pairs between non-neighboring groups.

The dynamic characteristics of imidazole–imidazole (or guanidine–guanidine) pairing can be studied using the same mathematical approach as for hydrogen bonding [81,82,83]. The structural relaxation $C_{pairs}\left(t\right)$ for imidazole–imidazole (or guanidine–guanidine) pairs (pair can break and then form again) defines the following time correlation function:

(14) $$C_{pairs}\left(t\right)=\frac{\langle b_{p}\left(0\right)b_{p}\left(t\right)\rangle }{\langle b_{p}\rangle }$$

where $b_{p}\left(t\right)$ is unity when a particular imidazole–imidazole (or guanidine–guanidine) pair forms at time *t*; otherwise, it is zero. The $C_{pairs}\left(t\right)$ are shown on Figure 13. We have extracted the relaxation time from each autocorrelation function ($\tau_{LF}$ from $C_{pairs}\left(t\right)$) for pairs between neighboring groups and for pairs between non-neighboring groups and presented them in Table 5). The main result is that neutral His–His pairs have the longest lifetimes. Taking into account the fact that the number of non-neighboring pairs is the largest among all possible pairs in the dendrimers studied, we can conclude that the pairing effect in the Lys–2His dendrimers with neutral histidines could have the greatest impact on the local and global properties of this particular dendrimers among all dendrimers studied by us.

### 3.4. The Interaction of Dendrimers with Molecules of Therapeutic Tetrapeptide

Dendrimers are often used to deliver drugs and genetic material to targeted cells, tissues and organs [8,9,10,12]. In this part of our article, we use the MD method to test the possibility of using the studied Lys–2His dendrimer as a delivery vehicle for small bioactive peptides in general, and for the tetrapeptide AEDG (Ala–Glu–Asp–Gly) in particular. This oligopeptide was synthesized to mimic the peptide drug “epithalamin” [85,86]. Each tetrapeptide molecule has a charge of −2. We assume that they should form a complex with the Lys-2His dendrimer at both normal and low pH since this dendrimer has positively charged terminal lysine groups with constant charge (+16) under these conditions. At the same time, this dendrimer has internal double histidine spacers that are neutral (2His, charge equal to 0) at normal pH and protonated (2Hisp, charge equal to +2) at low pH. All protonated spacers have the total charge equal to +28. Protonation leads to a significant increase in the total charge of the dendrimer (from +16 to +44) and to a change in its size, radial density and charge profiles. This should also lead to a change in its capacity for the transfer of oppositely charged drug molecules, for example, oligopeptides.

**Figure 14:**
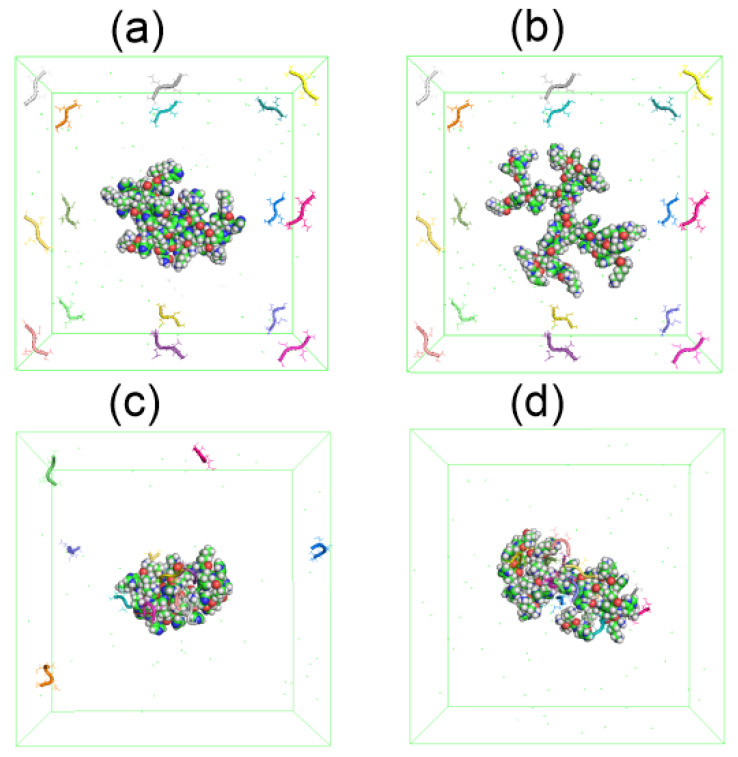
The snapshots of Lys–2His or Lys–2Hisp dendrimers and 16 AEDG peptides before the complex formation: (**a**) Lys2His+16AEDG and (**b**) Lys2Hisp + 16AEDG, and after the complex formation: (**c**) Lys2His + 16AEDG and (**d**) Lys2Hisp + 16AEDG.

We simulated a system consisting of one dendrimer at two different pH (Lys–2His or Lys–2Hisp, respectively) and 16 AEDG tetrapeptide molecules by the MD method under the same conditions as the dendrimer simulation described at the beginning of this article. At the beginning of the MD simulation, the dendrimer (Lys-2His or Lys-2Hisp) is located in the center of the periodic cubic simulation cell with a size of 9 nm, and 16 tetrapeptide molecules are located at the periphery of this cell (see Figure 14a,b). During the first half of the trajectory the peptides becomes closer and closer to each of dendrimer due to electrostatic interactions. However, at time 90–100 ns (see Figure 14c,d), all tetrapetides become completely adsorbed only by protonated Lys-2Hisp dendrimer while Lys-2His adsorbed only 11 of 16 tetrapeptides.

**Figure 15:**
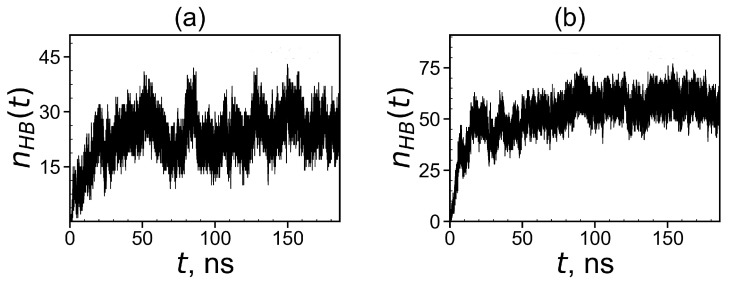
Time dependences of the number of hydrogen bonds $n_{hb}\left(t\right)$ between the Lys–2His or Lys–2Hisp dendrimers and 16 AEDG peptides during the complex formation: (**a**) Lys2His + 16AEDG and (**b**) Lys2Hisp + 16AEDG.

To demonstrate the formation of a complex between dendrimers and tetrapeptide molecules we calculated the instant number of hydrogen bonds $n_{hb}\left(t\right)$ between dendrimer and peptide molecules (see Figure 15). At the beginning of simulation in both systems, $n_{hb}\left(0\right)=0$ because free tetrapeptides are far from the dendrimer and, therefore, do not come into contact with it. When the peptides become closer to the dendrimer, contacts arise between them, and their number $n_{hb}\left(t\right)$ increases with time. Thus, the slope of $n_{hb}\left(t\right)$ during this initial time characterizes the rate of complex formation. It is clearly seen from these plots that the first system reaches dynamic equilibrium in about 60 ns. The second system reaches an equilibrium state after in 90 ns. From Figure 15, it follows that the average number of hydrogen bonds in the equilibrium state (at *t* > 60 ns) for the first complex is close to 24 and for the second complex in equilibrium (at *t* > 90 ns) it is close to 58.

**Figure 16:**
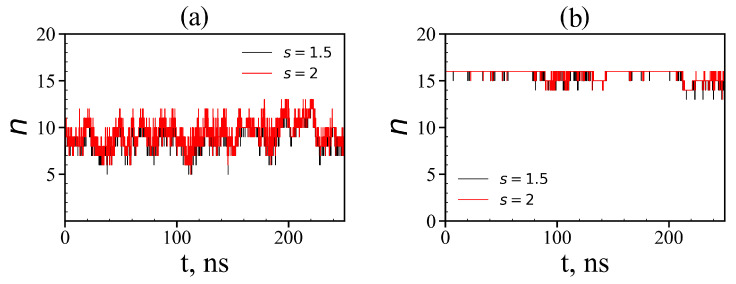
Time dependences of the number of tetrapeptides in complexes (local criteria): (**a**) Lys–2His + 16AEDG; (**b**) Lys–2Hisp + 16AEDG (two different value of distances s = 1.5 and 2 are used as the local criterion to define whether any atom of a given peptide molecule is close to any atom of the dendrimer or not).

After our systems reach equilibrium, we can calculate the equilibrium size and other characteristics of the complex. To do this, we need to know how many tetrapeptide molecules are in the complex with the dendrimer. For this goal we used a simple condition for a local criteria:

(15) $$r_{ij}<s\cdot r_{ij,min},$$

where $r_{ij}$ is the distance between the *i*-th atom of the dendrimer and the *j*-th atom of the peptide, $r_{ij,min}$ is the distance at which the potential of non-bonded interactions reaches a minimum for the corresponding types of atoms *i* and *j*, and *s* is a some factor (typically it is equal to 1.0, 1.5 and 2.0). If at least one atom of the peptide exists that satisfies the condition (15) with at least one atom of the dendrimer, then we assumed that such a peptide is complexed with the dendrimer. The instant numbers of tetrapeptide molecules in complexes with the Lys-2His and Lys-2Hisp dendrimers at different points in time are shown in Figure 16. It is easy to see that for the Lys-2His dendrimer this value fluctuates between 5 and 13, while for the Lys-2Hisp dendrimer it ranges from 13 to 16, but at most points in time, it is close to 16. The average value of the number of tetrapeptide molecules in the first complex is close to 9 and for the second complex it is close to 16 (see Table 6). Thus, the capacity of the Lys–2His dendrimer with neutral (2His) spacer to transport these tetrapeptide molecules is almost twice less than that of the Lys–2Hisp dendrimer with protonated (2Hisp) spacers.

**Figure 17:**
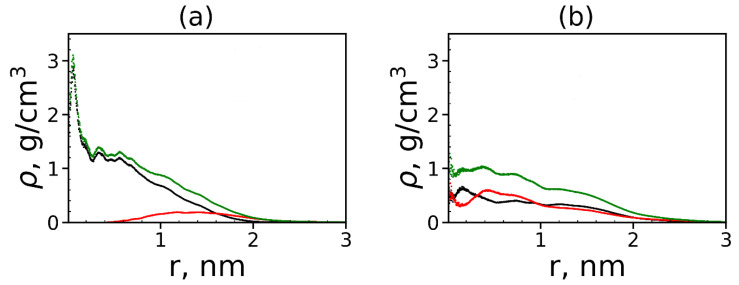
The radial distribution function of the density of: the dendrimer (black), peptide (red) atoms and all atoms in complexes (green) for (**a**) Lys–2His and (**b**) Lys–2Hisp dendrimers.

It is also interesting to know how the peptide molecules are distributed in complex: on the surface of each dendrimer or in its whole volume? To answer this question, we calculated the radial distribution functions of the atomic density of the dendrimer, peptides and all atoms in the complex (see Figure 17).

The size (the radius of gyration) of the first dendrimer with neutral histidine spacers in the complex is close to 1.2 nm (see Table 6). From Figure 17a, it is easy to see that most of the atoms of the tetrapeptide molecules (red curve) are located at a distance of more than r=1.2 nm from the center of the dendrimer. This means that tetrapeptides are manly located on the surface of the Lys–2His dendrimer and do not penetrate into it closer than r=0.5 nm from the center of the dendrimer. The size of the second dendrimer with protonated histidine spacers in the complex is ~1.6 nm but contrary to the first one, the density of tetrapeptide atoms increases in the center of the Lys–2Hisp dendrimer, and most of these atoms are inside this dendrimer (i.e., at a distance r<1.6 nm from the dendrimer center). This difference between the two complexes is due to the different distribution of charges in these dendrimers. In the Lys–2Hisp dendrimer with neutral 2His spacers, the positive charges are only in the terminal lysines (i.e., manly on the surface of the dendrimer). In the Lys–2Hisp dendrimer with protonated 2His spacers, the charges are distributed through the whole volume of the dendrimer.

## 4. Conclusions

Recently, it was shown that new lysine-based peptide dendrimer with Lys-2His repeating units is good candidate for siRNA delivery because it provides high transfection of siRNA to several cell lines [39]. NMR experiments [35,36] and MD simulations of the Lys–2Lys and Lys–2Arg dendrimers [40,41] were performed also. In this work, we performed the full atomic MD simulation of the Lys–2His peptide dendrimer at two conditions: at normal pH (when histidine residues are neutral and at low pH (when histidine residues are protonated). We compared the size and internal structure of both states of the Lys–2His dendrimer including the radial density and charge distribution in the dendrimer with neutral 2His spacers and the dendrimer with protonated 2Hisp spacers. We have shown that the dendrimer with neutral spacers is much more compact than the dendrimer with protonated spacers. The pairing of neutral His residues and protonated Hisp residues in these dendrimers was also studied and compared with the pairing of Arg residues. It was shown that the pairing of imidazole groups in the dendrimer with neutral His residues is stronger than in the dendrimer with protonated imidazole groups and guanidine groups of Arg residues in the Lys-2Arg dendrimer.

Interaction of Lys-2His and Lys-2Hisp dendrimer with bioactive AEDG peptide molecules and complex formation was studied also. In this article, we were interested in the use of histidine-containing dendrimers as nanocontainers for negatively charged bioactive peptide molecules. It was assumed that the more strongly positively charged Lys-2Hisp dendrimer would transfer them better, which was confirmed by simulation. We have shown that histidine-containing dendrimers could form complexes with AEDG tetrapeptides in both cases, but the capacity of the dendrimers is different. It was shown that the capacity of the Lys-2Hisp dendrimer with charged histidine spacers to delivery AEDG peptides is about twice higher than that of the Lys-2His dendrimer with neutral histidine spacers.

The higher hydrophobicity of the inner neutral part of the surface-charged dendrimer Lys-2His, leads to a more compact dendrimer state, and its effective cross-linking due to greater number of intradendrimer hydrogen bonds, and greater pairing effect between imidazole groups. Therefore, in the surface-charged dendrimer (Lys-2His), the molecules of this negatively charged tetrapeptide remain on the surface, while in the volume-charged dendrimer (Lys-2Hisp), they penetrate deep into the dendrimer. At the same time, as many drugs are hydrophobic, the hydrophobic inner part of the Lys-2His dendrimer can lead to better (than with the help of the Lys-2Hisp dendrimer) transfer of hydrophobic drug molecules. However, as both dendrimers are amphiphilic, to confirm this assumption it is necessary to carry out additional MD modeling, which we plan to carry out in the next article.

## Figures and Tables

**Table 1:** The characteristics of the Lys-2His and Lys-2Hisp dendrimers: the molecular mass of dendrimer *M* and its charge $Q_{bare}$, number $N_{end}$ and charge $Q_{end}$ of end groups, as well as number $N_{ins}$ and charge $Q_{ins}$ of inserted histidine amino acid residues and total number $N_{H_{2}O}$ of water molecules in systems, and the average size $a_{cell}$ of the simulation cell.

Dendrimer	*M* (g/mol)	$Q_{\mathrm{bare}}$ (e)	$N_{\mathrm{end}}$	$Q_{\mathrm{end}}$ (e)	$N_{\mathrm{ins}}$	$Q_{\mathrm{ins}}$ (e)	$N_{H_{2}0}$	$a_{\mathrm{cell}}$ (nm)
Lys-2His	5918.02	+16	16	+16	28	0	13,293	7.5
Lys-2Hisp	5946.24	+44	16	+16	28	+28	13,256	7.5

**Table 2:** The global characteristics of the Lys-2His and Lys-2Hisp dendrimers averaged through whole trajectory time: the radius of gyration $R_{g}$ (nm), the hydrodynamic radius $R_{h}$ (nm), the ratio $R_{h}/R_{g}$, the rigid-sphere approximation $\sqrt{5/3}R_{g}$ (nm), the position of terminal groups $R_{e}$ (nm), the effective radius $R_{max}$ (nm) (see subsection “Electrostatic interactions”) and shape anisotropy α at temperature 310 K.

Dendrimer	$R_{g}$	$R_{h}$	$R_{h}/R_{g}$	$\sqrt{5/3}R_{g}$	$R_{e}$	$R_{\max}$	α
Lys-2His	1.32	1.22	0.93	1.74	2.00	2.53	0.01
Lys-2Hisp	2.05	1.65	0.73	2.64	2.78	3.17	0.02

**Table 3:** The local characteristics of Lys-2His and Lys-2Hisp dendrimers: the average number of ion pairs $\langle n_{ion\;pairs}\rangle$ between counterions and charged groups in dendrimer, the effective dendrimer charge $Q^{*}$, the relative effective dendrimer charge $Q^{*}/Q_{bare}$, the surface charge density σ and ζ potential at the average body temperature T=310 K.

Dendrimer	$\langle n_{\mathrm{ion}\;\mathrm{pairs}}\rangle$	$\langle n_{\mathrm{ion}\;\mathrm{osmotic}}\rangle$	$Q^{*}$ (e)	$Q^{*}/Q_{\mathrm{bare}}$	σ (e/nm${}^{2}$)	ζ (mV)
Lys-2His	0.67	6.02	9.31	0.58	0.03	17.57
Lys-2Hisp	3.38	27.69	12.93	0.29	0.03	11.17

**Table 4:** The hydrogen bond characteristics of Lys-2His and Lys-2Hisp: the total average number of intra-dendrimer hydrogen bonds $\langle n_{H}^{id}\rangle$, the total average number of hydrogen bonds between the dendrimer and water $\langle n_{H}^{dw}\rangle$, the average number of hydrogen bonds between the side groups of spacers in the dendrimer and water ${\langle n_{H}^{dw}\rangle }_{side}$ and the average number of such hydrogen bonds per side group ${\langle n_{H}^{dw}\rangle }_{side}/N_{ins}$. The life-time of intra-dendrimer $\tau_{BF}^{id}$ and dendrimer–water $\tau_{BF}^{dw}$ hydrogen bonds at temperature T=310 K.

Dendrimer	$\langle n_{H}^{\mathrm{id}}\rangle$	$\langle n_{H}^{\mathrm{dw}}\rangle$	${\langle n_{H}^{\mathrm{dw}}\rangle }_{\mathrm{side}}$	${\langle n_{H}^{\mathrm{dw}}\rangle }_{\mathrm{side}}/N_{\mathrm{ins}}$	$\tau_{\mathrm{BF}}^{\mathrm{id}}$ (ps)	$\tau_{\mathrm{BF}}^{\mathrm{dw}}$ (ps)
Lys-2His	14.86	143.8	98.8	3.53	186.6	59.88
Lys-2Hisp	1.33	149.3	96.3	3.44	95.56	51.08

**Table 5:** The average number $n_{p}$ and the lifetime $\tau_{LF}$ (ps) of pairing at T=310 K for Lys–2His, Lys–2Hisp and Lys–2Arg.

	Lys-2His	Lys-2Hisp	Lys-2Arg
$n_{p}$			
neighbour	1.36	0.53	0.19
non-neighbour	15.04	1.59	3.7
$\tau_{LF}$			
neighbour	159.3	42.8	17.95
non-neighbour	104.2	33.06	33.58

**Table 6:** The radius of gyration of the Lys–2His (or Lys–2Hisp) dendrimer in complex, the radius of gyration of complex Lys–2His (or Lys–2Hisp) + 16AEDG and the number of peptide molecules in complex Lys-2His (or Lys-2Hisp) + 16AEDG. (Two parameters s = 1.5 and 2 were used to determine that any atom of the peptide is close to any atom of the dendrimer, and the results practically do not depend on the value of this parameter.)

	Lys-2His		Lys-2Hisp	
	s = 1.5	s = 2	s = 1.5	s = 2
$Rg_{dendrimer}$	1.22	1.22	1.57	1.57
$Rg_{complex}^{lc}$	1.46	1.47	1.63	1.63
$n^{lc}$	8.98	9.29	15.64	15.70
